# Temporal dystrophic remodeling within the intrinsic cardiac nervous system of the streptozotocin-induced diabetic rat model

**DOI:** 10.1186/2051-5960-2-60

**Published:** 2014-06-04

**Authors:** Chantalle E Menard, Melanie Durston, Elena Zherebitskaya, Darrell R Smith, Darren Freed, Gordon W Glazner, Ganghong Tian, Paul Fernyhough, Rakesh C Arora

**Affiliations:** University of Manitoba, Kragujevac, Manitoba Canada; St. Boniface Hospital/I.H. Asper Clinical Research Institute, CR3012 – 369 Tache Ave, Winnipeg, MB R2H 2A6 Canada

## Abstract

**Introduction:**

The pathogenesis of heart failure (HF) in diabetic individuals, called “diabetic cardiomyopathy”, is only partially understood. Alterations in the cardiac autonomic nervous system due to oxidative stress have been implicated. The intrinsic cardiac nervous system (ICNS) is an important regulatory pathway of cardiac autonomic function, however, little is known about the alterations that occur in the ICNS in diabetes. We sought to characterize morphologic changes and the role of oxidative stress within the ICNS of diabetic hearts. Cultured ICNS neuronal cells from the hearts of 3- and 6-month old type 1 diabetic streptozotocin (STZ)-induced diabetic Sprague-Dawley rats and age-matched controls were examined. Confocal microscopy analysis for protein gene product 9.5 (PGP 9.5) and amino acid adducts of (E)-4-hydroxy-2-nonenal (4-HNE) using immunofluorescence was undertaken. Cell morphology was then analyzed in a blinded fashion for features of neuronal dystrophy and the presence of 4-HNE adducts.

**Results:**

At 3-months, diabetic ICNS neuronal cells exhibited 30% more neurite swellings per area (p = 0.01), and had a higher proportion with dystrophic appearance (88.1% vs. 50.5%; p = <0.0001), as compared to control neurons. At 6-months, diabetic ICNS neurons exhibited more features of dystrophy as compared to controls (74.3% vs. 62.2%; p = 0.0448), with 50% more neurite branching (p = 0.0015) and 50% less neurite outgrowth (p = <0.001). Analysis of 4-HNE adducts in ICNS neurons of 6-month diabetic rats demonstrated twice the amount of reactive oxygen species (ROS) as compared to controls (p = <0.001).

**Conclusion:**

Neuronal dystrophy occurs in the ICNS neurons of STZ-induced diabetic rats, and accumulates temporally within the disease process. In addition, findings implicate an increase in ROS within the neuronal processes of ICNS neurons of diabetic rats suggesting an association between oxidative stress and the development of dystrophy in cardiac autonomic neurons.

## Introduction

The prevalence of diabetes mellitus is increasing at an alarming rate and parallel trends of the incidence of heart failure (HF) [1, 2]. It is estimated that the global number of people living with diabetes will reach 380 million by 2025 [3–6]. While the relationship between heart failure and diabetes can be partially explained by the concomitant risk factors associated with diabetes, evidence now supports diabetes as an independent risk factor for HF [4–9]. HF associated with diabetes mellitus, or “diabetic cardiomyopathy” has been defined to occur in the absence of both obstructive coronary artery disease (CAD) and hypertension [4, 7, 8, 10].

The correlation between diabetic cardiomyopathy (DC) and the development of HF is profound. The Framingham Study found the risk of developing HF to be two times higher in diabetic males, and five times higher in diabetic females, independent of hypertension, age, CAD, obesity and hyperlipidemia [2, 5, 6]. Additionally, diabetes has been used as a prognostic indicator in HF patients [11, 12], with the five-year mortality rate in patients with diabetic autonomic neuropathy having been reported to be as high as 53%, versus 15% with normal autonomic function [13–16]. The underlying pathogenesis of DC is only partially understood, but evidence suggests a link between autonomic dysfunction and abnormal myocardial function (heart rate and myocardial blood flow [10]) in diabetes mellitus.

Diabetic autonomic neuropathy is a significant clinical complication of diabetes that carries substantial patient mortality and morbidity [13, 14, 17, 18]. The parasympathetic and sympathetic nervous systems have been traditionally thought to operate in a simple relay fashion to control heart rate, but data now demonstrates that the mammalian heart possesses a complex nervous system, the intrinsic cardiac nervous system (ICNS), which can function independently to regulate the heart [19–21]. *In vivo* analysis of animal and human models have shown that intracardiac neuronal ganglia of the ICNS regulate cardiac output on a real-time, beat-to-beat basis, independent of extracardiac inputs (i.e. from the paravertebral ganglia) [19, 20, 22–24]. As such, the ICNS behaves as a potent neuronal modulator of cardiac function, and has been called the *“little brain on the heart”* [21, 25]. The ICNS contains afferent neurons [26, 27], sympathetic efferent postganglionic neurons [28–30], parasympathetic efferent postganglionic neurons [31], and a large population of local circuitry neurons that perform interconnections within the intrinsic cardiac ganglionated plexuses [28, 32]. The characterization of the neurons within the ICNS has been performed on both large [26, 29, 33–35] and small [36–39] animals, as well as the human heart [40–42]. Importantly, the human ICNS behaves in a very similar fashion to that observed in animal models [24, 43]. Given its importance, alteration of ICNS function is thought to play a central role in the pathophysiology of heart failure and arrhythmias [15, 44, 45].

Dystrophic changes in axonal and dendritic morphology, such as neurite swellings, aberrant neurite branching, and decreased neurite outgrowth, in the absence of significant neuronal loss, are the neuropathological hallmarks of diabetic neuropathy [18, 46–48]. The underlying process involved in the development of dystrophic and dysfunctional neurons has yet to be established, however it has been proposed that high serum glucose concentrations results in neurotoxicity with the eventual alteration or loss of nerve fibers [49, 50]. One possible culprit for the proposed neurotoxicity is an increased level of reactive oxygen species (ROS). Cultured adult rat dorsal root ganglion neurons (DRG) exposed to high physiological concentrations of glucose, results in an increased production of ROS, specifically within the neuronal axons, manifesting in axonal swelling and degeneration, but not in cell death [51]. Further evidence for ROS-mediated neurotoxicity was recently described by Campanucci and colleagues, who found that hyperglycemia induced a ROS-mediated depression of acetylcholine-evoked currents in the autonomic neurons of mice, thereby leading to a depression in synaptic transmission [52].

Previous reports of diabetic neuropathy have largely focused on extracardiac autonomic neurons. Examination of exposure of the isolated ICNS neurons to high glucose concentration has not been previously examined. We therefore sought to characterize the morphology of cultured ICNS in a diabetic rodent model. Secondly, we sought to examine the role of oxidative stress in the pathogenesis of neuronal dystrophy in the ICNS of a diabetic heart.

## Materials and methods

### Statement of ethics

The investigation conforms to the Guide for the Care and Use of Laboratory Animals published by the US National Institutes of Health (NIH Publication No. 85-23, revised 1996). All animal experiment protocols were approved by the University of Manitoba Animal Care Committee following the guidelines established by the Canadian Council on Animal Care.

### Primary cell culture

Primary ICNS cells were isolated from the hearts of 3-month (n = 5) and 6-month (n = 6) streptozotocin (STZ)-diabetic adult Sprague-Dawley male rats or age-matched control (n = 4 and n = 6, respectively). Rats were made type 1 diabetic with a single intraperitoneal injection of 75 mg/kg STZ (Sigma-Aldrich, St. Louise, MO). Body weight, plasma glucose and HbA1c was collected and used as endpoints for the development of diabetes (Table 1). Rats were deeply anesthetized with 5% isofluorane and euthanized by cervical decapitation. Hearts were rapidly removed and placed in cold 1xPBS. The atria was separated from the ventricles, the great vessels, and the atrial appendages, isolating the ICNS ganglionated plexus located within the dorsal fat of the central portion of the atria, near the pulmonary veins [38, 53, 54]. The isolated tissue was transferred to a dish containing cold filtered 1xPBS and then cut into small pieces. The tissue was then placed through two digestions; the first, consisting of 0.5% (w/v) collagenase (Worthington Biochemical, Lakewood, NJ) in 2 mL of warm F12 medium, and the second consisting of 0.25% (w/v) trypsin (Worthington) in 2 mL warm F12 medium. Both 1hr digestions were completed in a shaking incubator at 200 rpm and 37°C, with a wash of warm F12 performed in between. Following the second digestion, 1ml of fetal bovine serum (FBS) was added to deactivate the trypsin. 2 ml of F12 medium was added to the cell suspension and titrated using a smoothened Pasteur pipette. The supernatant was decanted into a sterile tube and this procedure was repeated, and the collected cell suspension was passed through a 70 μm cell filter into a sterile 50 ml falcon tube. The filtered cell suspension was then transferred to a sterile 15 ml falcon tube and centrifuged at 99.68 × g at room temperature (RT) for 5 minutes. The pellet was then re-suspended in 1 ml of F12 media, and added to a 1 ml solution of 15% BSA (O.5 ml F12 and 0.5 ml 30% BSA - Sigma-Aldrich, St. Louise, MO), which was then centrifuged at 99.68 × g for 10 minutes. The pellet was re-suspended in 1ml of growth medium (diabetic or control), and 150 μl of the cell suspension was added to the pre-treated cover slips in the 6-well cell culture plates. The plates were then placed in an incubator for 1 hour at 37°C and 5% CO_2_, after which point 1.5 ml of the appropriate growth medium was added to the wells, and the plates were returned to the incubator. Cells were visualized after 48 and 72 hours of growth using a phase contrast microscope (Nikon Eclipse TS100).

**Table 1:** **Characteristics of animal cohorts**

	Body weight (g)	Blood glucose (mmol/l)	HbA1c (%)
n	5	5	
Control	613.4 ± 58.5	8.3 ± 0.8	n.d.
Diabetic (3 mth)	382.2 ± 49.6*	34.0 ± 3.3*	n.d.
n	6	6	6
Control	760.7 ± 61.8	8.1 ± 0.8	3.6 ± 0.2
Diabetic (6 mth)	421.6 ± 52.5*	30.9 ± 3.3*	10.0 ± 2.1*

Values are means ± SD. Non-fasting blood glucose concentration was measured using the Accu-Chek Compact Plus glucometer (Roche, Laval, Quebec City, Canada) and blood glycated hemoglobin (HbA1c) levels by the A1CNow + system (Bayer Healthcare, Sunnyvale, CA). An asterisk (*) indicates statistically significant data with P ≤ 0.001 vs. control (using Student’s t-Test). N.d. = not determined.

### Preparation of cover slips

One day prior to cell isolation, sterile 22 mm coverslips (VWR, Mississauga, ON) were placed in 6-well culture plates and washed 3X with 2 ml of 1xPBS. The coverslips were coated in 1.5 ml of 0.5 mg/ml poly-DL-polyornithine (PORN) (Sigma-Aldrich, St. Louise, MO) and placed in a 37°C incubator overnight. On the morning of the isolation, the PORN was removed and the coverslips were washed with Ham’s F-12 nutrient mixture medium (F12 - Invitrogen, Carlsbad, CA). The coverslips were then coated with 1.5 ml of 1 μg/ml laminin (Nunclon Surface, Ottawa, ON, Canada) in F12 medium for 3 hours at a 37°C. The laminin was then removed and the cover slips washed 3X with F12 medium just prior to the addition of the cells.

### Preparation of growth medium

The growth medium used for neuronal culture consisted of Ham’s F12 medium, plus several additives. The neurotrophic factors were 0.3ng/ml of nerve growth factor beta (NGF-ß - Sigma-Aldrich, St. Louise, MO), 1 ng/ml of neurotrophin-3 (NT-3 - Sigma-Aldrich, St. Louise, MO), and 5 ng/ml of glial cell line-derived neurotrophic factor (GDNF - Sigma-Aldrich, St. Louise, MO). The stock N2 additive was prepared in F12 medium without antibiotic or insulin, and was composed of progesterone (Sigma-Aldrich, St. Louise, MO), putrescine (Sigma-Aldrich, St. Louise, MO), sodium selenite (Sigma-Aldrich, St. Louise, MO), fatty-acid free BSA powder (Sigma-Aldrich, St. Louise, MO), and transferrin (Sigma-Aldrich, St. Louise, MO). 40 μl of stock N2 was added per 4 ml of growth medium, bringing the final concentrations to 20 nM of progesterone, 100 μM of putrescine, 30 nM of sodium selinite, 0.1 mg/ml of BSA and 100 μg/ml of transferrin. To this standard growth medium (containing 10mM glucose), 0.1 nM of insulin was added, while a final concentration of 25 mM of glucose and zero insulin was used in the diabetic growth medium. It has been demonstrated that adult neurons do not undergo apoptosis under this high glucose preparation [52].

### Immunocytochemistry

After 72 hours in culture, the neurons were fixed with 4% paraformaldehyde in phosphate buffer at pH 7.4 for 15 min, followed by permeation with 0.3% Triton X-100 in PBS. Nonspecific binding was blocked by incubation with Blocking Reagent (Roche, Indianapolis, IN) combined with FBS and 1.0 M PBS in proportions of 3:1:1 for 1 hr at room temperature, followed by three washes with PBS. The coverslips were incubated in polyclonal antibody to amino acid adducts of (E)-4-hydroxy-2-nonenal (1:500 anti-4-HNE adducts Pab; Alexis Biochemicals, San Diego, CA) overnight at 4ºC in a humidified chamber. The next day, coverslips were washed several times in 1XPBS, followed by incubation in Alexa Flour 488 (Molecular Probes, Burlington, ON; 1:700) secondary antibody for 3 hr at room temperature. The coverslips were washed again in 1XPBS, and incubated with polyclonal anti-protein gene product (PGP) 9.5 (1:500 – UltraClone Limited, Isle of Wight, England) overnight at 4°C in a humidified chamber in the dark. The coverslips were then washed in 1XPBS, and then incubated for three hours with Alexa Fluor 546 (1:700). Coverslips were mounted to microscope slides using mounting medium with DAPI, Hard Set (Vector, Cat#H-1500). A Carl Zeiss LSM510 inverted confocal microscope was utilized for identification of neurons throughout specimens. Confocal images were captured using a Plan-Neofluar 40x/1.3 Oil DIC objective at 10 μm intervals ascending from the substrate though neuronal cell bodies and processes (Z sections). The images were reconstructed in three dimensions using Zeiss LSM Image Browser software (Germany).

### Morphometric analysis

**Figure 1:**
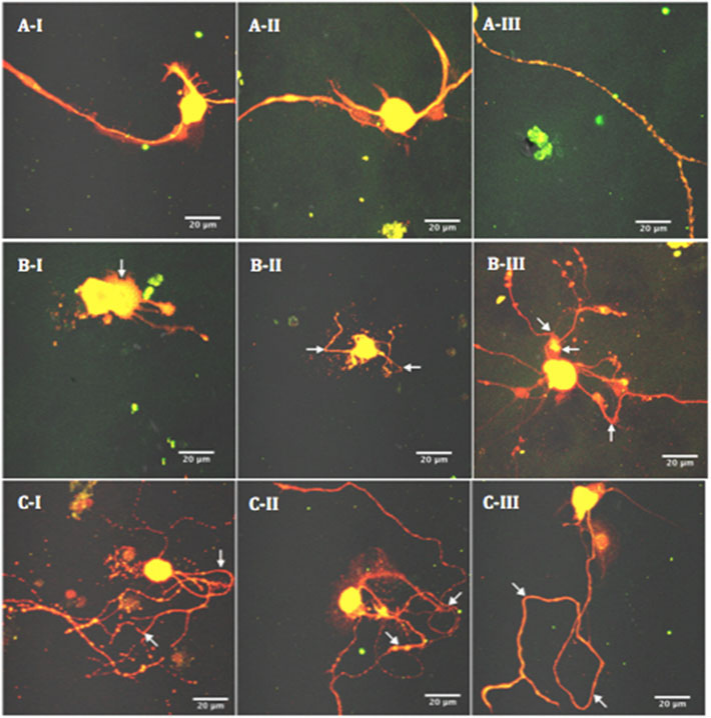
**Quantifying dystrophic features.** PGP-9.5 and 4-HNE co-stained neurons were classified into different categories, according to the degree of dystrophy, as defined by certain morphological features. Non-dystrophic neurons had a normal appearance, with smooth and intact processes **(AI-III)**. Mildly dystrophic neurons exhibited some features of dystrophy, such as outgrowths of lamellipodia-like structures from cell bodies and proximal neurites (arrows, **BI**), and sharp angles in neuronal processes (arrows, **BII** and **III**). Highly dystrophic neurons demonstrated irregular morphological features, such as aberrant neuritic growth from cell bodies and the appearance of frequent loops and curls in neuritic processes (arrows, **CI-III**).

The confocal images captured were analyzed in a blinded fashion to animal group. Two different methods of analysis were undertaken to determine the morphology of the neurons in culture. Firstly, dystrophic features quantified by assessment of the area of neurite outgrowth, and the number of neurite branches and swellings via ImageJ 1.42 software (http://rsbweb.nih.gov/ij/) on masked images [51, 55]. In brief, fluorescence images of PGP-9.5 and 4-HNE co-stained immunoreactive neurons were separated from their surroundings by a threshold value, the cell body was deleted from the image, and measurements were made to score the immunoreactive area within the pixel range [51, 55, 56]. Neurite swellings and branch points were then manually labeled and counted for each neuron. As specific staining was not done to differentiate axons from dendrites, all swellings observed on neuronal extensions will be referred to here as neurite swellings. Secondly, defined morphological features were used to categorize the neuronal cells into three different groups, according to the degree of dystrophy; non-dystrophic, mildly dystrophic and highly dystrophic [56] (Figure 1). Non-dystrophic neurons had a normal appearance, with smooth and intact processes (Figure 1A). Mildly dystrophic neurons exhibited some features of dystrophy, such as sharp angles in neuronal processes, and outgrowth of lamellipodia-like structures from cell bodies and proximal neurites (arrows, Figure 1B), while highly dystrophic neurons demonstrated highly irregular morphological features, such as aberrant neuritic growth from cell bodies and the appearance of frequent loops and curls in neuritic processes (arrows, Figure 1C) [56]. More than 200 neurons were scored per experimental condition.

### 4-HNE adduct analysis

The confocal images captured were analyzed with ImageJ 1.42 software in a blinded fashion. Fluorescence images of 4-HNE adduct staining immunoreactive neurons were separated from their surroundings by a threshold value, the cell body was deleted from the image, and measurements were made to score both the immunoreactive area within the pixel range and the intensity of the fluorescence. The average intensity of fluorescence was then divided by the average area of fluorescence.

### Data analysis

All data, with the exception of the dystrophic neuronal proportions, was subject to standard two-tailed unpaired Student’s *t*-Test with significance levels of p = 0.05, which does not assume equal variances, using GraphPad Prism 4 (GraphPad Prism 4, GraphPad Software Inc., San Diego, CA) [51]. All results are expressed as mean ± SEM. The data for the proportion of dystrophic neurons was compared using a normal approximation method (need references).

## Results

### Morphology of primary ICNS cultures

**Figure 2:**
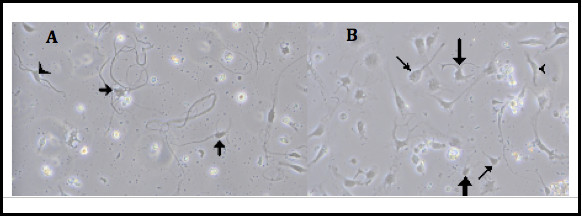
**Intrinsic cardiac neuron cultures.** Panels **A** and **B** are representative of normal neuronal cells in culture identified via phase contrast microscopy. Panel **A** demonstrates STZ-diabetic rat neurons at 48 hours in culture. Panel B demonstrates age-matched control rat neurons at 48 hours in culture. Several different neuronal types were isolated, including large pseudounipolar appearing cells similar to dorsal root ganglion cells (thin arrows), as well as bi- (arrowheads) and multi-polar (thick arrows) cells indicative of efferent or interneuronal cell types.

Phase contrast images (Figure 2) were taken of cultured STZ-diabetic and age matched control neurons at 72 hrs of culture. Neurons were assessed for morphology and confluence. As previously demonstrated in rodent ICNS cultures [57], three different neuronal types were found to have been isolated by evaluating cell morphology, including pseudounipolar neurons (cells with a large cell body and single long neurite), bipolar neurons (spindle-shaped cells with long neurites at opposite ends), and multipolar neurons (exhibiting several neurites of various lengths) (Figure 2).

### Effect of diabetes on neurite outgrowth

**Figure 3:**
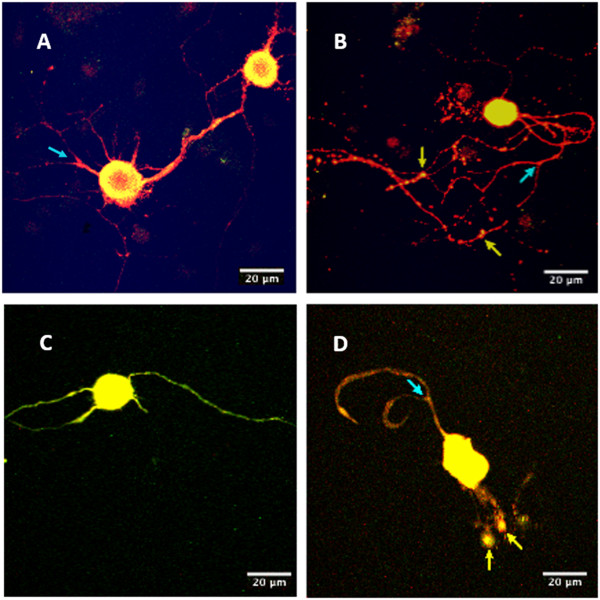
**Immunofluorescence of neuronal cultures.** Images were captured on a Carl Zeiss LSM510 inverted confocal microscope with a 40x objective. Panels **A** (age-matched control) and **B** (STZ-diabetic) are images collected from the 3-month rat cultures of PGP-9.5 and 4-HNE co-stained neurons, while panels **C** (age-matched control) and **D** (STZ-diabetic) are images of the PGP-9.5 and 4-HNE co-stained neurons from the 6-month rat cultures. The dystrophic feature of neurite swelling (yellow arrows) is indicated in panels **B** and **D**, and examples of neurite branching (blue arrows) can be seen in panels **A**, **B** and **D**.

**Figure 4:**
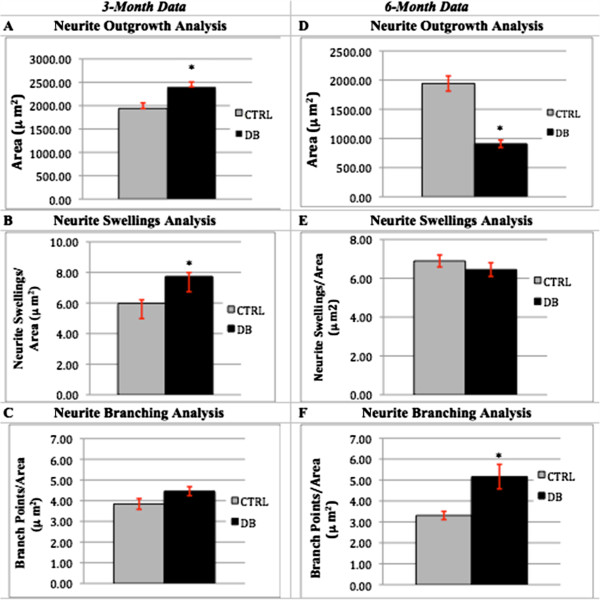
**Neurite outgrowth, neurite swellings and neurite branching as measures of dystrophy.** Panels **A**, **B**, and **C** represent the data collected from 3-month control (n = 101) and diabetic (n = 128) rat neuronal cultures. An asterisk (*) indicates statistically significant data. Error bars indicate the mean ± the S.E.M. At 3 months, area (neuronal outgrowth) and the number of neurite swellings were both found to be significantly increased in diabetic neurons, at, respectively, 1.2X (p = 0.0042) and 1.3X (p = 0.01) that of control neurons. Branching was higher in diabetic neurons, although not significantly, at 1.16X the control, p = 0.0688. Panels **D**, **E**, and **F** represent the data collected from 6-month control (n = 129) and diabetic (n = 108) rat neuronal cultures. At 6 months, area was found to be significantly lower in the diabetic neurons, as compared to the control neurons, at 0.48X (p = <0.001). While there was no significant difference between diabetic or control neurons in the amount of neurite swellings (p = 0.3438), the diabetic neurons were seen to have a significantly higher amount of neurite branching, at 1.56X (p = 0.0015) that of the control neurons.

**Figure 5:**
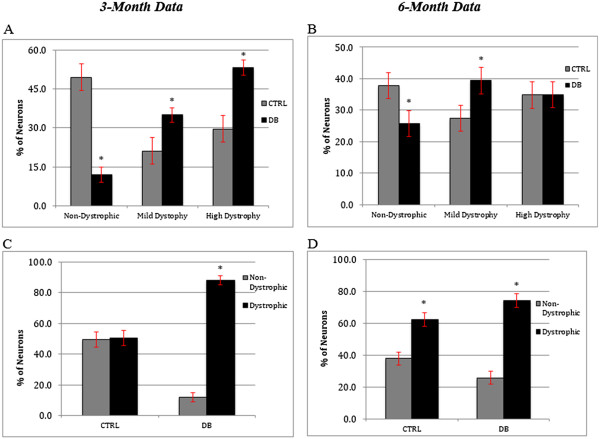
**Morphological assessment of neuronal dystrophy.** Panels **A** and **C** represent data collected from the 3-month neuronal cultures. As panel A indicates, at 3 months, there were significantly higher proportions of mildly (34.9% vs. 21.1%, p = 0.0244) and highly dystrophy (53.2% vs. 29.5%, p = 0.0004) neurons in the diabetic neurons vs. the control neurons. Panel C indicates there was a significantly higher proportion of any dystrophy (mild or high) in the diabetic vs. the control neurons (88.1% vs. 50.5%, p = <0.0001). Panels **B** and **D** represent data collected from 6-month neuronal cultures. At 6 months, there was a significantly higher proportion of mild dystrophy in diabetic neurons vs. control (39.4% vs. 27.4%, p = 0.008), but no statistical significance to the proportions of neurons that were highly dystrophic. Additionally, as seen in Panel D, there was a significantly higher proportion of any dystrophy (mild or high) in the diabetic neurons vs. the controls (74.3% vs. 62.2%, p = 0.0448). Error bars indicate the mean ± the S.E.M.

Cultures of 3- and 6-month diabetic rat neurons co-stained with PGP-9.5 and 4-HNE (Figure 3) were examined for neurite outgrowth, neurite swellings and neurite branching. At 3-months, STZ-diabetic rat culture ICNS neurons demonstrated a 20% increase in the area of neurite outgrowth (Figure 4A; p = 0.0042), and 30% more neurite swellings per area (Figure 4B; p = 0.01) as compared to controls. However, neurite branching was not significantly different between culture groups (Figure 4C). At 6 months, cultured STZ-diabetic rat neurons demonstrated a decrease in the area of neurite outgrowth (Figure 4D), at only 50% of the control neurons (p = <0.001). The difference in the number of neurite swellings was not found to be statistically significant (Figure 4E), however cultured diabetic rat ICNS neurons exhibited a significant increase in neurite branching as compared to controls (Figure 4F), with 50% more branch points per area (p = 0.0015). Furthermore, at 3-months, cultured STZ-diabetic rat ICNS neurons demonstrated a greater proportion of any dystrophic features compared to control ICNS neurons, with higher proportions of both mildly dystrophic (34.9% vs. 21.1%, p = 0.0244) and highly dystrophic (53.2% vs. 29.5%, p = 0.004) neurons (Figure 5A). There was also a higher proportion of any dystrophy (mild or high) in the diabetic neurons as compared to controls (88.1% vs. 50.5%, p = <0.0001; Figure 5C). At 6-months, cultured STZ-diabetic rat ICNS neurons were also found to have higher proportions of mildly dystrophic neurons (39.4% vs. 27.4%, p = 0.008; Figure 5B). There was no difference in the proportion of highly dystrophic neurons at 6 months, however, a higher proportion of any dystrophy (mild or high) in diabetic neurons as compared to control neurons was observed (74.3% vs. 62.2%, p = 0.0448; Figure 5D).

### Expression of 4-HNE adducts

**Figure 6:**
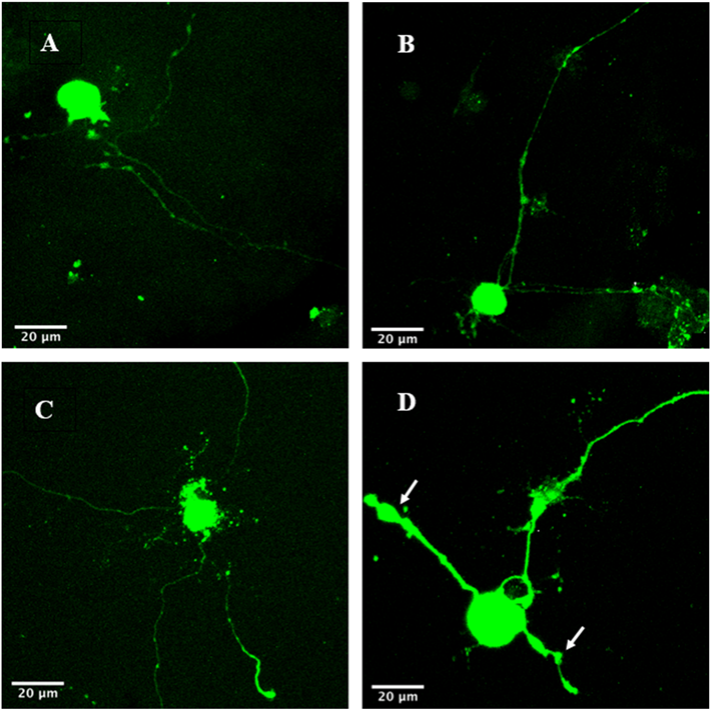
**Immunofluorescence of 4-HNE adducts.** Panels **A** (age-matched control) and **B** (STZ-diabetic) are images of 3-month neuronal cultures. Panels **C** (aged-matched control) and **D** (STZ-diabetic) are images of 6-month neuronal cultures. Neurons were stained and imaged using immunofluorescence for 4-HNE adducts. Images were captured on a Carl Zeiss LSM510 inverted confocal microscope with a 40x objective. The software program ImageJ 1.42 (available at http://rsbweb.nih.gov/ij/) was used to quantify both the intensity of fluorescence, and the total neurite area staining positive for 4-HNE adducts. Note in panel D the increased intensity of fluorescence expressed in the neurite swellings (white arrows).

**Figure 7:**
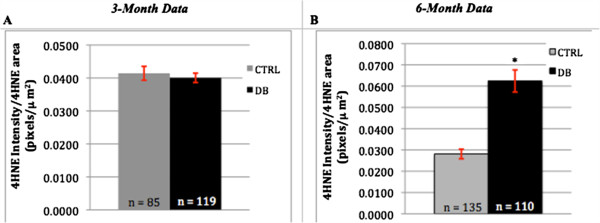
**Intensity of 4-HNE adduct staining.** Panels **A** and **B** are the graphical representation of the fluorescence intensity recorded for 4-HNE adduct staining vs. the total 4-HNE staining neurite area for both 3- and 6-month STZ-diabetic and age-matched control rat neuronal cultures. An asterisk (*) indicates statistically significant data. Error bars indicate the mean ± the S.E.M. At 3 months (panel **A**), there was no statistical significance in 4-HNE adduct staining between diabetic and control neurons (p = 0.5755). At 6 months (Panel **B**), there was a significant difference observed, with the diabetic neurons displaying a 4-HNE adduct staining intensity per total 4-HNE staining area that was 2.22X that seen in the controls (p = <0.0001).

Cultures of 3- and 6- month diabetic rat neurons were stained for the amino acid adducts of 4-HNE as a marker of ROS production (Figure 6). At 3 months, diabetic and control intrinsic cardiac neurons demonstrated no differences in the intensity of staining for 4-HNE adducts (Figure 7A). However, by 6 months, cultured intrinsic cardiac neurons from the diabetic heart demonstrated a significant increase in intensity of 4-HNE adducts as compared to that of the control neurons ((p = <0.001; Figure 7B).

## Discussion

This study demonstrated that isolated intrinsic cardiac neurons from adult STZ-diabetic and age-matched control rats can be maintained in culture, and that ICNS neurons show varied morphology, indicative of the cell types that exist *in vivo* within the cardiac ganglia. It was further demonstrated that neuronal dystrophy occurs in the ICNS neurons of STZ-induced diabetic rats, and accumulates temporally within the disease process. In addition, an increase in 4-HNE adducts occurs in the neuronal processes implicating an association between oxidative stress and the development of a dystrophy in cardiac autonomic neurons. At the time of writing this manuscript, we are not aware of any previous study examining the effect of high-glucose on the cardiac autonomic nervous system.

Analysis of the 3-month data indicated that only one quantifiable dystrophic feature, the number of neurite swellings, was significantly greater in the diabetic ICNS neurons. Neurite swellings, thought to be the residues of aberrant intra-ganglionic sprouting, may be axonal or dendritic in nature, and have been described as being large accumulations of small mitochondria and neuronal structural proteins [18, 46, 47, 51]. The significance of neurite swellings in the pathogenesis of neuronal dysfunction may be two-fold. Firstly, the collection of debris may reflect or induce alterations in axonal transport [55, 58]. Altered axonal transport may result in death of mitochondria and the absence of structural proteins at distal sites, and the distal axon, lacking the sufficient tools for plasticity, is predisposed to dissolution upon subsequent injury or stress [18, 51]. Secondly, the collection of mitochondria themselves may produce a local exaggeration of oxidative stress via the overproduction of superoxide by the electron transport chain in the mitochondria, leading to a self-propagating pathway of neuritic dystrophy [18, 59]. In accordance with this view, the neurite swellings exhibited a greater level of 4-HNE adduct expression (Figure 6).

At 6-months, an increase in the number of dystrophic features was observed in the diabetic ICNS neurons, with two quantifiable dystrophic features (area of neuronal outgrowth and neurite branching) being significantly different from controls. Increased neurite branching has been postulated as a compensatory response of a neuron to a loss of synapses occurring along their length [60, 61]. An aberrant increase in branching morphology has also been observed in neurons affected with Alzheimer’s disease (AD), characterized by a pattern of ineffective intensive growth [61]. Given the parallels that have been drawn between the effects of AD and diabetes on neurons, it is possible that this type of neurite branching may also be occurring in diabetic neurons [62–64]. Furthermore, increased neurite branching has been described in diabetic rat neurons of prevertebral and paravertebral sympathetic ganglia [48]. In diabetic neurons, the development of axonal sprouts appears to serve as a substrate for further dystrophic changes, with their appearance preceding the development of axonal swellings [48, 65]. At 6 months, the decrease in area of neuronal outgrowth as compared to controls is an expected and often observed finding in dystrophic diabetic neurons, owing to the degeneration of axons and nerve fiber loss [46, 58, 66]. The number of neurite swellings did not differ significantly between the 6-month diabetic and control neuronal groups.

Overall, analysis of 3-month and 6-month data demonstrated an increase in the dystrophic features in the diabetic neurons between the two time points, with two of the dystrophic features being more prominent in the 6-month diabetics, compared to only one feature for the 3-month diabetics. Importantly, it has been demonstrated in other diabetic rat peripheral neurons that the dystrophic effects of diabetes accumulate temporally, with dysmorphology progressing from mild at early time points to more pronounced at later time points [66]. Given the temporal relationship between our two experimental groups, our data is in agreement with these findings.

Due to the observed tortuosity in the neuronal processes, we performed a confirmatory analysis of dystrophic phenotype for the ICNS neurons. The classification of neurons into dystrophic or non-dystrophic categories was undertaken so as to compare neurons as a whole [56]. Importantly, at both time points, there was a significantly higher proportion of any dystrophy (mild or high) in the diabetic neurons. That the 6-month diabetic group did not exhibit a significantly greater proportion of highly dystrophic neurons was not an expected result, but a possible explanation is that age itself produces similar dystrophic changes in neuronal morphology [67].

Several studies have confirmed staining for the presence of 4-HNE adducts as a marker for oxidative stress [51, 52, 55, 68, 69]. 4-HNE adducts are produced via oxidative stress dependent-lipid peroxidation [52], and it has been proposed that oxidative stress plays an important role in the formation of diabetes-associated dystrophic neuronal features [55, 68]. Given the significant increase in the intensity of staining for 4-HNE adducts in the 6-month diabetic neurons, our study provides evidence to support this hypothesis. As the 3-month diabetic neurons did not show a difference in staining intensity compared to the control group, this speaks to the aforementioned increase in dystrophy that is seen to occur temporally in diabetes [66]. As stated previously, another important finding in our study was the increased intensity of staining for 4-HNE adducts that was observed inside the neurite swellings (Figure 6), which supports the notion that oxidative stress is associated with the formation of dystrophic features in diabetic neurons [55, 59].

This data adds to the growing body of evidence supporting the physiologic importance of ICNS in the diabetic heart. A recent study by Mabe and Hoover [70] illustrated that a functional parasympathetic nervous system cannot negate the deleterious effects of a dysfunctional ICNS. Using a STZ-mouse model, they demonstrated a reduction in resting heart rate, as well as alterations with high and low frequency power (via spectral analysis of electrocardiograms), in diabetic mice, suggesting a relative reduction of parasympathetic tone. Further confirmation via immunohistochemical analysis demonstrated no loss of cardiac cholinergic neurons or nerve fibers, nor altered cholinergic sensitivity of the atria. The authors thus concluded that the observed heart rate abnormalities in the diabetic mice may be as a result of defective ganglionic neurotransmission. The dysmorphic alterations observed in our analysis of diabetic ICNS neurons supports their proposition that the true neuronal dysfunction lies within the intracardiac ganglia.

We acknowledge that a limitation of our analysis is the lack of *in vivo* functional correlation to the morphologic changes observed in the *in vitro* data presented. This may have best been achieved with the use of some *in vivo* assessment, such as echocardiography or other real-time imaging modality. While we unfortunately did not have access to this technology at the time of the study, other investigators have demonstrated a correlation with STZ-induced DM in rodents and left ventricular systolic and diastolic dysfunction, fibrosis and abnormal SERCA2a activity [71, 72]. In addition, previous work examining the alterations of the intrinsic cardiac nervous system in disease states, in both animal [73–76] and human models [24], have demonstrated functional remodeling of ICNS neurotransmission to have profound effects on cardiodynamics.

A second limitation of our study is the inability to draw causality between the increase in 4-HNE staining and ROS-induced toxicity. To further elucidate the role of ROS in neuronal dysmorphology and dysfunction, experimentation with anti-oxidants, as well as electrophysiology studies should be undertaken. Additional work will also be necessary to further characterize the observed neurite swellings as axonal or dendritic, and to confirm the contents of the neurite swellings. Furthermore, our study did not address the role of this ROS-induced toxicity on neuronal cell viability. Future studies investigating cell viability and apoptosis would be helpful to determine whether neuronal dysfunction is also the result of neuronal cell loss in the intracardiac ganglia.

## Conclusion

Our study demonstrated for the first time in ICNS neurons of STZ-diabetic rats that dystrophic features accumulate temporally in diabetic neurons, and that features associated with neuronal oxidative stress are observed concurrently with the development of dystrophy. This study also challenged the notion that the measurement of total area of neuronal outgrowth is a sufficient measure of neuronal outgrowth. Given the observed tortuosity of neuronal processes, additional methods for neurite outgrowth assessments, such as the length of the longest axon, might be more informative. While this data provides the inference of ROS as a mechanism of morphologic changes that occur in the ICNS when exposed to high-glucose concentrations, research is required to confirm that oxidative stress plays a causative role in dystrophic changes, and to evaluate whether the dystrophic changes observed in diabetic rat ICNS neurons leads to neuronal dysfunction.

## Availability of supporting data

The data set(s) supporting the results of this article is (are) included within the article (and its additional file(s).

## Abbreviations

HF: Heart failure
ICNS: Intrinsic cardiac nervous system
STZ: Streptozotocin
4-HNE: E-4-hydroxy-2-nonenal
CAD: Coronary artery disease
DC: Diabetic cardiomyopathy
ROS: Reactive Oxygen Species
DRG: Dorsal Root Ganglion
FBS: Fetal bovine serum
RT: Room temperature
PORN: Poly-DL-polyornithine
PGP: Polyclonal anti-protein gene product
AD: Alzheimer’s disease.
